# Exonic Deletions and Deep Intronic Variants of the *SLC26A4* Gene Contribute to the Genetic Diagnosis of Unsolved Patients With Enlarged Vestibular Aqueduct

**DOI:** 10.1155/2024/8444122

**Published:** 2024-10-15

**Authors:** Yongan Tian, Mengli Liu, Yu Lu, Xiaoyan Zhao, Zhiqiang Yan, Yi Sun, Jingyuan Ma, Wenxue Tang, Haili Wang, Hongen Xu

**Affiliations:** ^1^Precision Medicine Center, Academy of Medical Science, Tianjian Laboratory of Advanced Biomedical Sciences, Zhengzhou University, Daxuebei Road No. 40, Zhengzhou 450052, China; ^2^The Research and Application Center of Precision Medicine, The Second Affiliated Hospital of Zhengzhou University, Jingba Road No. 2, Zhengzhou 450014, China; ^3^Department of Otolaryngology-Head and Neck Surgery, West China Hospital of Sichuan University, Chengdu 610041, China; ^4^Institute of Rare Diseases, West China Hospital of Sichuan University, Chengdu 610041, China; ^5^Department of Otolaryngology Head and Neck Surgery, Hospital of the 71st Group Army/Affiliated Huaihai Hospital of Xuzhou Medical University, Xuzhou 221004, China; ^6^Department of Otolaryngology Head and Neck Surgery, General Hospital of Central Theater Command, Wuhan 430070, China; ^7^Department of Otolaryngology, Henan Provincial People's Hospital of Zhengzhou University, Zhengzhou 450003, China; ^8^Longhu Laboratory, Zhengzhou University, No. 100, Science Avenue, Zhengzhou 450001, China; ^9^National Health Commission Key Laboratory of Birth Defects Prevention, Henan Key Laboratory of Population Defects Prevention, Henan Institute of Reproduction Health Science and Technology, Zhengzhou 450002, China

**Keywords:** copy number variations, enlarged vestibular aqueduct, intronic variants, multiplex PCR, *SLC26A4*

## Abstract

Enlarged vestibular aqueduct (EVA) is a frequently occurring inner ear malformation that associates with sensorineural hearing loss (SNHL), with *SLC26A4* being the responsible gene. Based on multiplex PCR enrichment and sequencing of the exonic and flanking regions of the *SLC26A4* gene, we developed a panel specifically for EVA and found that up to 95% of EVA patients in our Chinese cohorts carried biallelic *SLC26A4* pathogenic variants (M2). In this study, we tried to investigate the genetic etiology of 13 previously undiagnosed EVA patients with monoallelic (M1) or none (M0) *SLC26A4* variant using a stepwise approach, including copy number variation (CNV) analysis of multiplex PCR enrichment and next-generation sequencing data, single-molecule real-time (SMRT) sequencing of the whole *SLC26A4* gene, whole exome sequencing (WES), and whole genome sequencing (WGS). CNV analysis revealed deletions in Exons 1–3, Exons 5–6, and Exons 9–10 of the *SLC26A4* gene in seven patients, and SMRT sequencing identified the same heterozygous deep intronic variant (NM_000441.2:c.304+941C>T) in two patients, resulting in a final diagnosis in 9/13 patients. Notably, the variants of Exons 9–10 deletion and c.304+941C>T have not been reported previously. We further showed that the variant c.304+941C>T led to the exonization of partial AluSz6 element (126 bp) where the variant is located through sequencing of the mRNA extracted from the blood of a heterozygous variant carrier. In conclusion, our stepwise approach improved the diagnosis rate of EVA, expanded the mutational spectrum of the *SLC26A4* gene, and highlighted the contribution of exonic deletions and deep intronic variants to EVA.

## 1. Introduction

Enlarged vestibular aqueduct (EVA) (OMIM: 600791) is the most prevalent inner ear malformation among children with sensorineural hearing loss (SNHL) [1]. EVA can manifest as a nonsyndromic condition, such as autosomal recessive deafness 4 (DFNB4), or as part of complex syndromes like Pendred syndrome [2, 3]. Pendred syndrome was characterized by EVA, goiter (thyroid gland enlargement), and severe congenital HL when it was first discovered [4]. The HL associated with DFNB4 and Pendred syndrome is fluctuating or progressive, with onset occurring pre, peri, or even postlingually, and the severity and laterality are highly variable [5].

Both DFNB4 and Pendred syndrome are inherited as autosomal recessive caused by biallelic pathogenic variants of the *SLC26A4* gene (solute carrier family 26, member 4). Although other genes, including *FOXI1* [6] and *KCNJ10* [7], have been linked to EVA, some studies have raised doubt regarding their involvement in EVA [8]. Additionally, digenic inheritance of *SLC26A4* and *EPHA2* in Pendred syndrome has been proposed and confirmed in two Japanese EVA families [9]. Pathogenic variants of *SLC26A4* are one of the most common causes of hereditary HL worldwide [10]. In China, *SLC26A4* is the second most common causal gene in the population with HL, accounting for 13%–22% of patients [11–13].

Copy number variation (CNV) generally refers to the increase or decrease of the copy number of genomic fragments longer than 1 kb, which is one of the important factors leading to human diseases [14]. It has been suggested that CNVs are a common cause of nonsyndromic HL [15]. Therefore, CNVs of *SLC26A4* may also be an important cause of EVA. Up to now, several types of *SLC26A4* CNVs have been reported, including deletions of Exons 1–2 [16], Exons 1–3 [17, 18], Exons 4–6 [19], Exons 5–6 [20], Exon 7 [15], Exon 8 [21], and Exons 11–18 [16] (Table S1). We and others have demonstrated that up to 95% of Chinese EVA patients can be diagnosed by sequencing the exonic and flanking regions of the *SLC26A4* gene [8, 22, 23]. However, there are still some EVA patients who cannot be diagnosed after genetic testing, which is a great challenge for genetic counseling.

**Figure 1:**
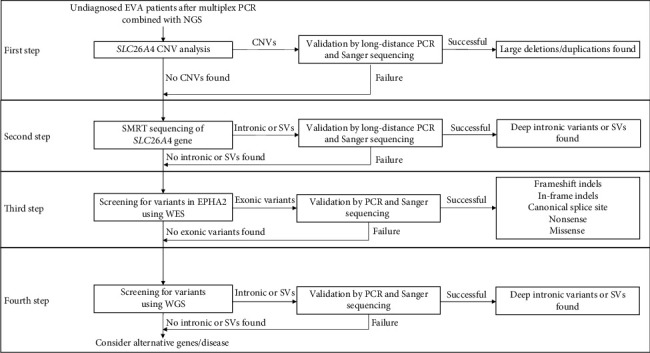
The stepwise approach of genetic testing used in this study.

Here, we used a stepwise genetic testing strategy including CNV analysis, single-molecule real-time (SMRT) sequencing, whole exome sequencing (WES), and whole genome sequencing (WGS) for patients who were undiagnosed (M0 or M1) after multiplex PCR enrichment combined with next-generation sequencing (NGS) of the exonic and flanking regions of *SLC26A4* (Figure 1). Exonic deletions and deep intronic variants were identified in the majority of patients (9/13, 69%), leading to a final diagnosis.

## 2. Materials and Methods

### 2.1. Inclusion Criteria and Clinical Evaluation

We previously developed a multiplex PCR panel for diagnosing EVA patients by targeting the exonic and flanking regions of the *SLC26A4* gene [23]. By only considering single nucleotide variants (SNVs) and small indels in exonic and flanking regions, the genetically undiagnosed EVA patients with monoallelic (M1) or non-*SLC26A4* variants (M0) were considered to be enrolled in the study. The physical examination of patients and the criteria for EVA were the same as those described in our previous study [23]. The inclusion criteria were as follows: (1) probands diagnosed with EVA, (2) probands with M0 or M1 found by the multiplex PCR panel, (3) probands with both parental samples available, (4) probands with no syndromic features except goiter, (5) probands with no drug-induced HL, and (6) probands with Han Chinese ancestry from Henan Province, China. According to the 2021 World Health Organization (WHO) classification of HL, the mean HL at 0.5, 1, 2, and 4 kHz of better hearing ear was classified into five tiers: mild (20–< 35 db HL), moderate (35– < 50 db HL), moderately-severe (50– < 65 db HL), severe (65– < 80 db HL), profound (80– < 95 db HL), and deafness (≥ 95 db HL).

All subjects volunteered to participate in this project and signed an informed consent form (subjects under the age of 18 were signed by their guardians). The experimental protocol was approved by the Medical Ethics Committee of the Second Affiliated Hospital of Zhengzhou University (Approval No. 2018008).

### 2.2. Exonic CNV Analysis of Multiplex PCR Enrichment and NGS Data

To identify potential genetic causes in families that remained undiagnosed after multiplex PCR combined with NGS followed by SNV and small indel analysis, we extracted sequencing results from both the probands and their parents. We then utilized the mosdepth software [24] to analyze the average sequencing depth of each amplified region in each sample, with the goal of identifying any abnormal amplification in specific regions. To accomplish this, we normalized the average sequencing depth of each amplified region by assuming a total data amount of 10 Mbp for each sample. We compared the average sequencing depth of each amplified region across samples to identify any regions with unequal amplification. Samples with either high or low sequencing depth were suspected to have large deletions or duplications in the corresponding region. Families suspected of having CNVs were further analyzed and verified using long-distance PCR and SMRT sequencing.

### 2.3. Long-Distance PCR and Sanger Sequencing

We designed primers for the entire region of the *SLC26A4* gene using NCBI Primer-blast software and had them synthesized by Shanghai Shangya Biotechnology Co., Ltd. Specific primers and Takara RT-PCR Kit were used to amplify the targeted region, and the amplified products were analyzed using 1% agarose gel electrophoresis to verify the length of the amplified products and check for nonspecific amplification. After the amplified products were purified, Sanger sequencing was performed using Applied Biosystems.

### 2.4. PacBio SMRT Sequencing

PacBio SMRT targeted sequencing of six genes (including *GJB2*, *SLC26A4*, *GJB3*, *GJB6*, *POU3F4*, and *MT-RNR1*) was developed and performed by Grandomics (Beijing, China), and it maybe served as the first-layer test for hearing loss (HL) patients as it includes common HL genes in China. To cover the six genes, DNA probes of 120 bases were designed and synthesized by Boke Biotechnologies (Boke, Beijing, China). The PacBio SMRT sequencing library was constructed using a Template Prep Kit (PacBio, Menlo Park, United States) according to the instructions. Three micrograms of genomic DNA was sheared to 1~6 kb fragments by a g-Tube (#520079; Covaris, Bankstown, Australia) centrifugation. An SMRTbell library was prepared and sequenced on Pacific Biosciences with the Sequel II platform.

### 2.5. WES and WGS

The implementation steps of WES, WGS, bioinformatics analysis, and variant interpretation were performed as described in our previous studies [12, 23, 25]. The WES data were analyzed to identify variants in the coding regions of EVA-associated genes, including *SLC26A4*, *FOXI1*, *KCNJ10*, and *EPHA2*. Additionally, the WGS data were examined for any potentially overlooked coding or intronic variants, as well as variants located in the *cis*-regulatory elements of the *SLC26A4* gene, with regulatory regions defined in accordance with [26].

### 2.6. Functional Consequence Analysis of Deep Intronic Variants

Five millimeters of peripheral blood was extracted from a healthy donor and the father (a heterozygous carrier for the intronic variant c.304+941C>T) of Patient 3312305-1 using density gradient centrifugation to separate peripheral blood mononuclear cells (PBMC). Total RNA from PBMC was extracted following the instructions of the RNA extraction kit (Sparkjade, China). The concentration and quality of the RNA were measured using NanoDrop One. Subsequently, cDNA was synthesized through reverse transcription using the extracted RNA as a template. PCR was conducted using cDNA and the specified primers 5′-CTTTCCAGCAACAGCACGAG-3′ and 5′-CACTGGAAAAGGTCCAACTGAGA-3′. The PCR products were analyzed by 1% agarose gel electrophoresis to determine the molecular weight. After that, the desired bands were quickly excised and purified. The purified target fragments were then cloned into the pUCm-T vector (Beyotime, China), and Sanger sequencing was performed using universal primers M13R: 5′-CAGGAAACAGCTATGAC-3′.

### 2.7. Variant Classification

The variant classification was performed according to the guidelines of the American College of Medical Genetics and Genomics (ACMG) for the interpretation of sequence variants [27] and the ClinGen Hearing Loss Expert Group's specific recommendations [28].

## 3. Results

### 3.1. Clinical Features and Genetic Prescreening

A total of 13 probands with undiagnosed EVA were included in this study. From the previous strategy of multiplex PCR combined with NGS [23], monoallelic variants of *SLC26A4* were detected in nine of the probands, while four had no variant in the *SLC26A4* gene. All probands presented with bilateral sensorineural HL, and the onset age ranged from 0 to 15 years old. The degree of HL was severe to profound, and five patients showed stable HL, seven patients were progressive, and one patient was fluctuating (Table 1).

### 3.2. Identification and Verification of CNVs in *SLC26A4* Exonic Regions

We conducted CNV analysis of the *SLC26A4* gene based on the NGS results of 13 undiagnosed EVA probands (as described in Materials and Methods), of which seven patients were found to harbor suspected CNVs in coding regions. The heterozygous exonic deletions detected in this step included (Table 2): Exons 1–3 deletion in Patients 3312024-1, 3312061-1, 3312180-1, and 3312757-1; Exons 5–6 deletion in Patient 3312177-1; and Exons 9–10 deletion in Patient 3312189-1. We also detected homozygous Exons 5–6 deletion in Patient 3312285-1. The homozygous deletions or the compound heterozygosity of heterozygous deletions combined with previously detected SNVs (see Table 3 for variant classification) of the *SLC26A4* gene could explain the clinical phenotypes of these seven patients.

**Figure 2:**
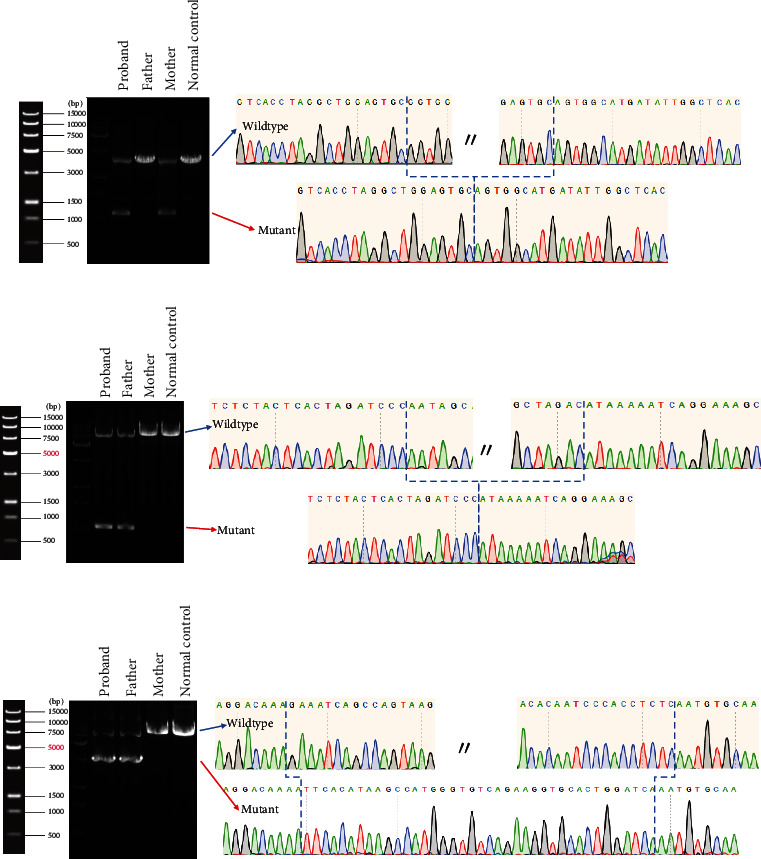
Exonic deletions (Exons 5–6, Exons 1–3, and Exons 9–10) from representative probands and families. (a) Agarose gel electrophoresis and Sanger sequencing of Exons 5–6 deletion (1845 bp) in the Family 3312177. The upper image of the Sanger sequencing shows the proband and the lower image shows the proband's mother. The red arrows indicate the breakpoint. (b) Agarose gel electrophoresis and Sanger sequencing of Exons 1–3 deletion (7666 bp) in the Family 3312180. The upper image of the Sanger sequencing shows the proband and the lower image shows the proband's father. The red arrows indicate the breakpoint. (c) Agarose gel electrophoresis and Sanger sequencing of Exons 9–10 deletion (4979 bp) in the Family 3312189. The upper image of the Sanger sequencing shows the proband and the lower image shows the proband's father. The red arrows indicate the breakpoint.

To determine the breakpoints for the above exonic deletions in the *SLC26A4* gene, we designed primers and performed long-distance PCR. The results of agarose gel electrophoresis confirmed the presence of the above CNVs (Figure 2). Sanger sequencing and further SMRT sequencing validation of probands revealed the length of deletions in Exons 1–3 (7666 bp, chr7:107300016-107307681, GRCh37), Exons 5–6 (1845 bp, chr7:107314217-107316062), and Exons 9–10 (4979 bp, chr7:107329303-107334282) (Figure 2 and Figure S1ABC). It was noteworthy that SMRT sequencing of 3312757-1 revealed a shorter version of Exons 1–3 deletion (deletions of Exons 1–2 and partial Exon 3) (3152 bp, Figure S1D). The results obtained from the SMRT sequencing validation step confirmed the accuracy and reliability of our approach.

### 3.3. Deep Intronic Variants Detected by SMRT Sequencing

**Figure 3:**
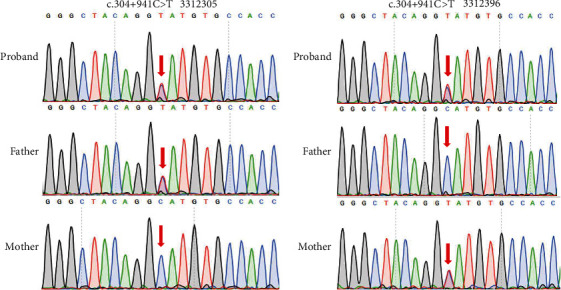
Sanger sequencing verification of *SLC26A4* c.304+941C>T in the Families (a) 3312305 and (b) 3312396.

To identify potential genetic causes for the remaining six patients who were undiagnosed, we conducted SMRT sequencing of the entire *SLC26A4* gene to detect structural and intronic variants. While no structural variants were found, we did discover a deep intronic variant (NM_000441.2:c.304+941C>T) in Patients 3312305-1 and 3312396-1. Sanger sequencing confirmed that Patient 3312305-1 inherited the variant from her father (Figure 3(a)) and Patient 3312396-1 inherited from her mother (Figure 3(b)). Therefore, both patients were diagnosed as compound heterozygosity of a deep intronic variant with a previously detected SNV (Tables 2 and 3).

In order to validate the variants c.304+941C>T and investigate its contribution to the HL cohorts, we screened the variant using whole-genome sequencing data in the China Deafness Genetics Consortium (CDGC) cohort enrolling 20,666 unrelated HL cases. We totally found five EVA patients harboring c.304+941C>T and other known *SLC26A4* pathogenic variants, including c.919-2A>G, c.281C>T, and c.1226G>A, accounting for 0.24‰ of the CDGC cohort (Table S2) (see Table 3 for variant classification).

### 3.4. Deep Intronic Variant c.304+941C>T Results in Exonization of Partial Alu Element

The varSEAK software (https://varseak.bio/) predicted this variant to be a Class 3 variant of unknown splicing effect and SpliceAI predicted this variant might affect splicing (SpliceAI Acceptor Gain delta score: 0.51 and SpliceAI Donor Gain delta score: 0.29) [42].

**Figure 4:**
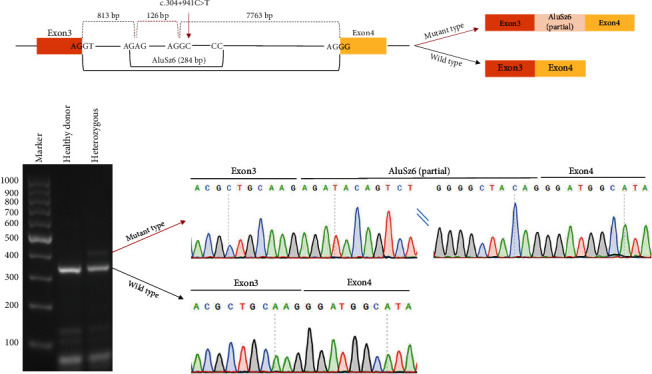
Functional consequence of *SLC26A4* deep intronic variant c.304+941C>T. (a) Schematic diagram of the location of the variant c.304+941C>T relative to AluSz6 element and surrounding *SLC26A4* exons and its functional consequence. (b) Left: gel electrophoresis of amplification cDNA product for peripheral blood mononuclear cell mRNA from a healthy donor (wildtype for c.304+941C>T) and the father (heterozygous for c.304+941C>T) of patient 3313305-1. Right: Sanger sequencing of two bands showed in the left panel.

To determine if the deep intronic variant c.304+941C>T affects splicing, we reverse-transcribed total RNA isolated from PBMC of a normal individual and a heterozygous carrier and amplified the exons flanking the variant. Gel electrophoresis showed that compared to a normal individual, the heterozygous mutant individual displayed two bands: one corresponding to wild-type cDNA and the other to mutant cDNA. The Sanger sequencing of mutant cDNA revealed that the deep intronic variant c.304+941C>T created a novel splicing site between *SLC26A4* Exons 3 and 4. This resulted in the inclusion of the mRNA of the 126 bp sequence, which is partial to the AluSz6 element (Figure 4).

### 3.5. No Variants Detected by WES and WGS

Despite our previous efforts, four patients remained undiagnosed. To explore potential genetic causes, we employed WES and WGS to search for pathogenic variants in regulatory regions for the *SLC26A4* gene or in other genes (for example, *FOXI1*, *KCNJ10*, and *EPHA2*) known to be associated with EVA. However, we did not identify any pathogenic variants in related genes or in the regulatory regions of the *SLC26A4* gene in the four samples.

## 4. Discussion

In this study, we performed a comprehensive genetic analysis on 13 undiagnosed EVA patients. Our approach included CNV analysis of multiplex PCR enrichment and NGS data, SMRT sequencing, WES, WGS, and Sanger sequencing. By CNV analysis, we detected deletion variants in Exons 1–3, Exons 5–6, and Exons 9–10 in 7 patients, which were validated by long-distance PCR and Sanger sequencing. In addition, SMRT sequencing led to a final diagnosis in two patients with deep intronic variant c.304+941C>T affecting splicing. However, four patients remained undiagnosed after WES and WGS were used to detect variants in EVA-associated genes other than *SLC26A4*. Our stepwise testing strategy expanded the mutational spectrum of the *SLC26A4* gene and improved the diagnosis rate of EVA in China.

The diagnostic rate of EVA patients varies significantly in different races. Previous studies indicated that *SLC26A4* biallelic variants were detected in one to three-quarters of EVA patients in Caucasian populations [43, 44]. In EVA cohorts from different regions of China, biallelic *SLC26A4* variants were found in 65%–95% of patients [23, 45]. In this study, we identified exonic CNVs in 7 out of 13 previously undiagnosed EVA patients, while deep intronic variants were detected in 2 patients, accounting for 69% (9/13) of the cohort in total. In previous studies, pathogenic SNVs and small indels in the coding region were mainly focused [2], while exonic CNVs and deep intronic variants were less frequently detected in EVA patients. Although the recently identified haplotype, called Caucasian EVA (CEVA), seems to be responsible for EVA in the majority of patients with monoallelic variants [26, 46], the underlying genetic etiology of the undiagnosed EVA patients' needs to be further explored. The present study confirms that exonic CNVs and deep intronic variants play a significant role in undiagnosed EVA patients, which provides new possibilities for the diagnosis of Caucasian and Chinese EVA patients. Single-molecular sequencing for the whole *SLC26A4* gene may provide a solution for these patients in searching for pathogenic variants in exonic, intronic, and regulatory regions, as it has proven effective in detecting structural variants in hereditary HL [47, 48].

The transcription of eukaryotic DNA into mature mRNA requires the excision of introns from precursor RNA, and genetic and cellular changes that disrupt the accuracy of splicing processes can lead to various human diseases [49]. Studying aberrant splicing patterns in these diseases helps us understand the underlying problems with splicing regulation in pathological conditions. One class of splicing defect is the activation of a cryptic splice site (or related cryptic exonization mediated by transposable elements), which causes the retention of a segment or an element of transposable element within an intron [49]. The deep intronic variant c.304+941C>T identified in this study creates new splice sites and leads to the exonization of the partial Alu element, which represents this class of splicing defect. Alu elements, a primate-specific subclass of short interspersed nuclear elements, make up 11% of the human genome, with more than one million copies located mainly in intronic and intergenic regions [50]. The insertion of Alu elements into genes may disrupt or create novel functions by affecting RNA polyadenylation, editing, and splicing [51]. The Alu exonization, in which an intronic Alu element is spliced in as a new exon into a transcript, can lead to the production of novel protein isoforms by introducing a premature termination codon that triggers the nonsense–mediated decay mechanism to degrade aberrant transcripts [51, 52]. In the present study, the inclusion of 126 bp AluSz6 element will cause an in-frame insertion of 42 amino acids, which is a known pathogenic mechanism for the *SLC26A4* gene although it is relatively rare [53]. In addition, a number of intronic variants have been reported in patients with HL, including c.254-649T>G (*CLRN1*) [54] and c.705+3767_705+3768del (*PCDH15*) [55], further highlighting the contribution of intronic variants to HL.

In conclusion, for undiagnosed EVA patients after multiplex PCR combined with NGS, a stepwise approach adopted in this study saved time in analyzing complex WGS data, alleviated the financial burden of patients, and improved the diagnosis rate of EVA. The identification of novel variants, including the Exons 9–10 deletion and c.304+941C>T, has expanded the mutational spectrum of the *SLC26A4* gene, which will contribute to the prevention and management of hereditary HL. However, it should be noted that some patients in this study remain undiagnosed, and their pathogenic variants may have been missed by this stepwise approach. Additionally, there may be other as yet unrecognized genes that cause EVA. Nevertheless, with the advent of SMRT sequencing, the declining cost of NGS, and the increasing understanding of noncoding and regulatory regions, it is expected that the genetic etiology of these patients will be revealed in the near future.

## Figures and Tables

**Table 1:** Clinical phenotypic features of 13 previously undiagnosed probands.

**Patient ID**	**Gender**	**Test age (years)**	**Onset age (years)**	**Degree of HL**	**Evolution of HL**
3310066-1	M	8	0	Profound	Stable
3310615-1	F	3	0	Profound	Progressive
3312024-1	M	3	1	Profound	Progressive
3312051-1^a^	M	1	0	Severe	Stable
3312061-1^a^	M	33	15	Severe	Progressive
3312177-1	M	4	0	Severe to profound	Progressive
3312180-1	M	3	0	Severe	Fluctuating
3312189-1	M	8	1	Profound	Progressive
3312285-1	M	7	1	Profound	Stable
3312305-1	F	2	0	Profound	Progressive
3312396-1	F	6	0	Profound	Stable
3312635-1	M	2	0	Profound	Stable
3312757-1	F	5	2	Severe to profound	Progressive

^a^Probands from our previous study [23].

**Table 2:** Genotypes of 13 previously undiagnosed probands revealed by a stepwise approach.

**Patient ID**	**SLC26A4 genotype (based on NM_000441.2)**	
	**Paternal allele**	**Maternal allele**
3310066-1		
3310615-1		
3312024-1	c.279T>A	Exons 1–3 deletion (7666 bp)
3312051-1		
3312061-1	c.1264-6T>G	Exons 1–3 deletion (7666 bp)
3312177-1	c.919-2A>G	Exons 5–6 deletion (1845 bp)
3312180-1	Exons 1–3 deletion (7666 bp)	c.1667A>G
3312189-1	Exons 9–10 deletion (4979 bp)	c.1614+1G>A
3312285-1	Exons 5–6 deletion (1845 bp)	Exons 5–6 deletion (1845 bp)
3312305-1	c.304+941C>T	c.946G>T
3312396-1	c.1315G>A	c.304+941C>T
3312635-1	c.2168A>G	
3312757-1	Exons 1–3 deletion (3152 bp)	c.919-2A>G

**Table 3:** Pathogenicity classification of variants identified in the study based on ACMG guidelines.

**Variants**	**ACMG criteria**	**Classification**
c.279T>A, p.(Ser93Arg)	PM2: not found in the gnomAD database. PS3: Experimental studies have shown that this variant affects *SLC26A4* protein function [29]. PM3_VeryStrong: pathogenic variants confirmed in trans in four patients and phase unknown in four patients [8, 29–31]. PP4: patient's phenotype highly specific for the gene.	Pathogenic

c.281C>T, p.(Thr94Ile)	PM2: gnomAD genomes East Asian allele frequency = 0.00005437 < 0.00007. PM3_VeryStrong: Pathogenic variant confirmed in trans in one patient (this study) and phase unknown in six patients [8]. PP3: REVEL score > 0.7. PP4: patient's phenotype highly specific for gene.	Pathogenic

c.304+941C>T	PM2: gnomAD genomes allele frequency = 0.00001594 < 0.00007. PS3_Moderate: Validated functional studies show a deleterious effect (this study). PM3_VeryStrong: pathogenic variant confirmed in trans in seven patients (this study). PP4: patient's phenotype highly specific for gene.	Pathogenic

c.946G>T, p.(Gly316Ter)	PVS1: null variant in the gene with established LOF as a disease mechanism. PM2: not found in the gnomAD database. PM3_VeryStrong: pathogenic variants confirmed in trans in two patients and phase unknown in six patients [8, 30, 32]. PP4: patient's phenotype highly specific for gene.	Pathogenic

c.1264-6T>G	PM2: not found in the gnomAD database. PP3: Variant is predicted to affect splicing: dbscSNV ADA score = 0.993789. PM3: pathogenic variants confirmed in trans in one patient (this study). PP4: patient's phenotype highly specific for gene.	Likely Pathogenic

c.1315G>A, p.(Gly439Arg)	PM2: gnomAD genomes allele frequency = 0.00002 < 0.00007. PM3_Strong: pathogenic variant confirmed in trans in two patients [33, 34]. PP3: REVEL score > 0.7. PP4: patient's phenotype highly specific for gene.	Likely Pathogenic

c.1547dup	PVS1: null variant in the gene with established LOF as a disease mechanism. PM2_Supporting: gnomAD East Asian allele frequency = 0.000272 < 0.0007. PM3_VeryStrong: pathogenic variant confirmed in trans in two patients and phase unknown in five patients [30, 35–37]. PP4: patient's phenotype highly specific for gene.	Pathogenic

c.1614+1G>A	PVS1: null variant in the gene with established LOF as a disease mechanism. PM3: pathogenic variant confirmed in trans in two patients (this study). PM2: gnomAD genomes allele frequency = 0.00001 < 0.00007. PP4: patient's phenotype highly specific for gene.	Pathogenic

c.1667A>G, p.(Tyr556Cys)	PM2: gnomAD genomes allele frequency = 0.00001594 < 0.00007. PM3_Strong: homozygous variant confirmed in four patients and phase unknown in two patients [38–40]. PP3: REVEL score > 0.7. PP4: patient's phenotype highly specific for gene.	Pathogenic

chr7:107,300,016–107,307,681 Exons 1–3 deletion (7666 bp)	PVS1: null variant in the gene with established LOF as a disease mechanism. PM2: not found in the gnomAD database. PM3_VeryStrong: pathogenic variant confirmed in trans in more than four patients [17, 18, 41]. PP4: patient's phenotype highly specific for gene.	Pathogenic

chr7:107,300,698–107,303,849 Exons 1–3 deletion (3152 bp)	PVS1: null variant in the gene with established LOF as a disease mechanism. PM2: not found in the gnomAD database. PM3: pathogenic variant confirmed in trans in one patient (this study). PP4: patient's phenotype highly specific for gene.	Pathogenic

chr7:107,314,217–107,316,062 Exons 5–6 deletion (1845 bp)	PVS1: null variant in the gene with established LOF as a disease mechanism. PM2: not found in the gnomAD database. PM3_VeryStrong: pathogenic variant confirmed in trans in four patients [20] (this study). PP4: patient's phenotype highly specific for gene.	Pathogenic

chr7:107,329,303–107,334,282 Exons 9–10 deletion (4979 bp)	PVS1: Null variant in the gene with established LOF as a disease mechanism. PM2: not found in the gnomAD database. PM3: pathogenic variant confirmed in trans in one patient (this study). PP4: patient's phenotype highly specific for gene.	Pathogenic

## Data Availability

The data of this study are available from the corresponding authors upon reasonable request.

## Nomenclature

CNV: copy number variation
DFNB4: autosomal recessive deafness 4
EVA: enlarged vestibular aqueduct
HL: hearing loss
NSHL: nonsyndromic hearing loss
SMRT Sequencing: single molecule real-time sequencing
SNHL: sensorineural hearing loss
SNV: single nucleotide variant
WES: whole exome sequencing
WGS: whole genome sequencing
WHO: World Health Organization

## Supporting Information

Supporting Information Additional supporting information can be found online in the Supporting Information section. Table S1. Reported exon deletions in the *SLC26A4* gene. Table S2. Genotypes of five additional EVA patients harboring the c.304+941C>T variant from the China Deafness Genetics Consortium (CDGC) cohort. Figure S1. SMRT sequencing validation of probands revealed the length of deletions in Exons 1–3 (A), Exons 5–6 (B), Exons 9–10 (C), and a short version of Exons 1–3 deletion (D).
