# Supplementary material for: Exonic Deletions and Deep Intronic Variants of the SLC26A4 Gene Contribute to the Genetic Diagnosis of Unsolved Patients With Enlarged Vestibular Aqueduct
Source: Hum Mutat. 2024 Oct 15;2024:8444122. doi: 10.1155/2024/8444122 (PMC11919234; doi:10.1155/2024/8444122)
Supplement: Supporting Information — Additional supporting information can be found online in the Supporting Information section. Table S1. Reported exon deletions in the SLC26A4 gene. Table S2. Genotypes of five additional EVA patients harboring the c.304+941C>T variant from the China Deafness Genetics Consortium (CDGC) cohort. Figure S1. SMRT sequencing validation of probands revealed the length of deletions in Exons 1–3 (A), Exons 5–6 (B), Exons 9–10 (C), and a short version of Exons 1–3 deletion (D). [file 8444122.f1.pdf]

Supplemental Table 1. Reported exon deletions in the *SLC26A4* gene.

Exon Deletions	Length	Clinical phenotype	References
exons 1–2	24368bp	Pendred syndrome	[1]
exons 1-3	7666bp	EVA	[2-4]
exons 4-6	14052bp	Pendred syndrome	[5]
exons 5-6	1.8kb	EVA	[6]
exon 7	NA	Pendred syndrome	[7]
exon 8	NA	Pendred syndrome	[8]
exons 11-18	11202bp	Pendred syndrome	[1]

[1] S. Anwar, et al., "SLC26A4 mutation spectrum associated with DFNB4 deafness and Pendred's syndrome in Pakistanis," *J Hum Genet*, vol. 54, no. 5, pp. 266-270, 2009.

[2] Y. H. Lin, et al., "Targeted Next-Generation Sequencing Facilitates Genetic Diagnosis and Provides Novel Pathogenetic Insights into Deafness with Enlarged Vestibular Aqueduct," *J Mol Diagn*, vol. 21, no. 1, pp. 138-148, 2019.

[3] X. Pang, et al., "A 7666-bp genomic deletion is frequent in Chinese Han deaf patients with non-syndromic enlarged vestibular aqueduct but without bi-allelic SLC26A4 mutations," *Int J Pediatr Otorhinolaryngol*, vol. 79, no. 12, pp. 2248-2252, 2015.

[4] Y. Liu, et al., "Application value of high-throughput gene copy number variation detection in the diagnosis of enlarged vestibular aqueduct," *Zhonghua Yi Xue Za Zhi*, vol. 101, no. 2, pp. 103-107, 2021.

[5] A. Pera, et al., "A mutational analysis of the SLC26A4 gene in Spanish hearing-impaired families provides new insights into the genetic causes of Pendred syndrome and DFNB4 hearing loss," *Eur J Hum Genet*, vol. 16, no. 8, pp. 888-896, 2008.

[6] H. Hu, et al., "Molecular analysis of hearing loss associated with enlarged vestibular aqueduct in the mainland Chinese: a unique SLC26A4 mutation spectrum," *J Hum Genet*, vol. 52, no. 6, pp. 492-497, 2007.

[7] A. E. Shearer, et al., "Copy number variants are a common cause of non-syndromic hearing loss," *Genome Med*, vol. 6, no. 5, pp. 37, 2014.

[8] C. M. Sloan-Heggen, et al., "Comprehensive genetic testing in the clinical evaluation of 1119 patients with hearing loss," *Hum Genet*, vol. 135, no. 4, pp. 441-450, 2016.

Supplemental Table 2. Genotypes of five additional EVA patients harboring the c.304+941C>T variant from the China Deafness Genetics Consortium (CDGC) cohort

**Patient ID**	**SLC26A4 genotype (based on NM_000441.2)**	
	**Paternal Allele**	**Maternal Allele**
HL-011525	c.304+941C>T	c.1547dup
HL-012438-1	c.919-2A>G[a]	c.304+941C>T
HL-000347	c.919-2A>G	c.304+941C>T
HL-010478	c.304+941C>T	c.281C>T
HL-013071	c.304+941C>T	c.1226G>A

[a] the paternal sample was not available, and c.919-2A>G was assumed from the father.

Supplemental Figure 1. SMRT sequencing validation of probands revealed the length of deletions in exons 1-3 (A), exons 5-6 (B), exons 9-10 (C), and a short version of exons 1-3 deletion (D).

(A) Exons 1-3 deletion (7,666 bp)

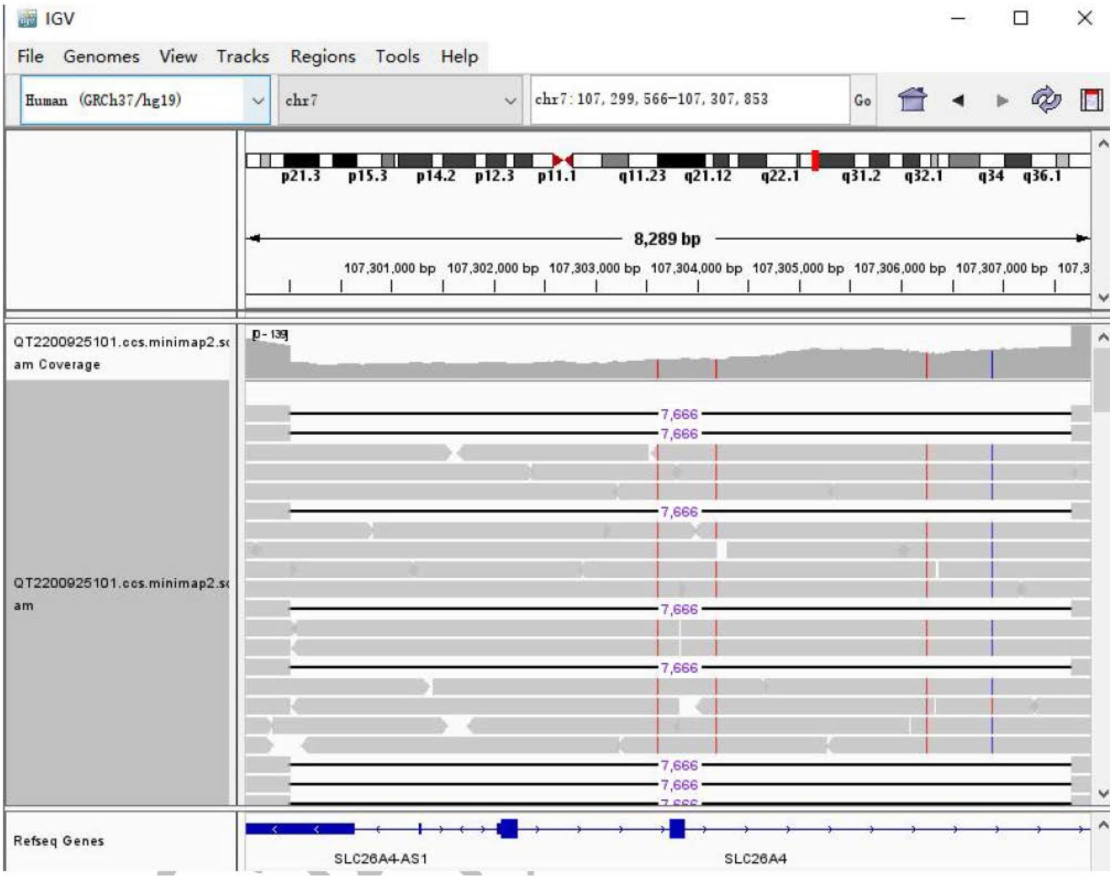

(B) Exons 5-6 deletion (1,845 bp)

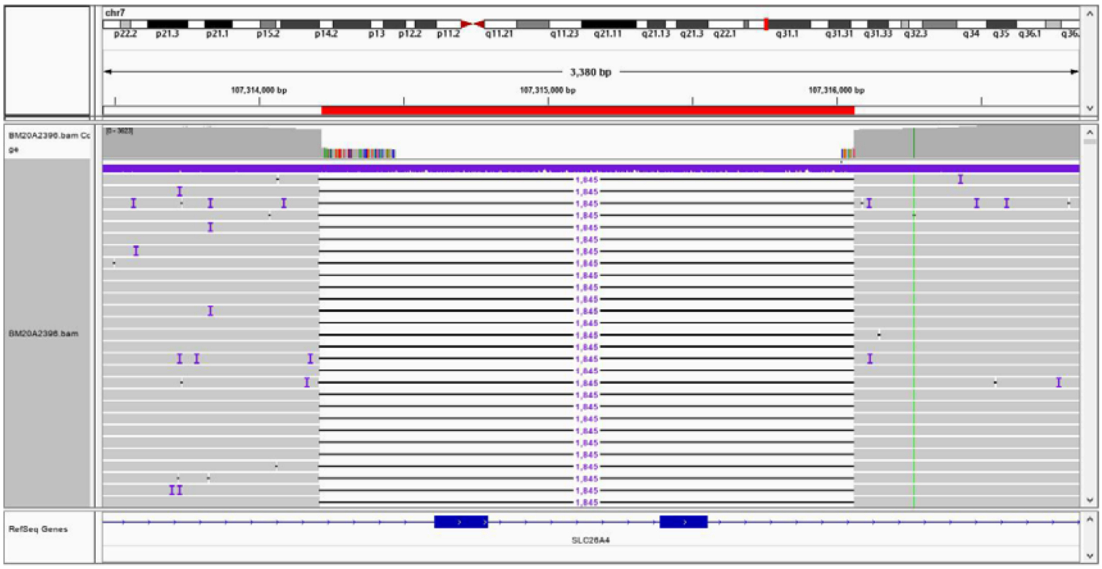

(C) Exons 9-10 deletion (4,979 bp)

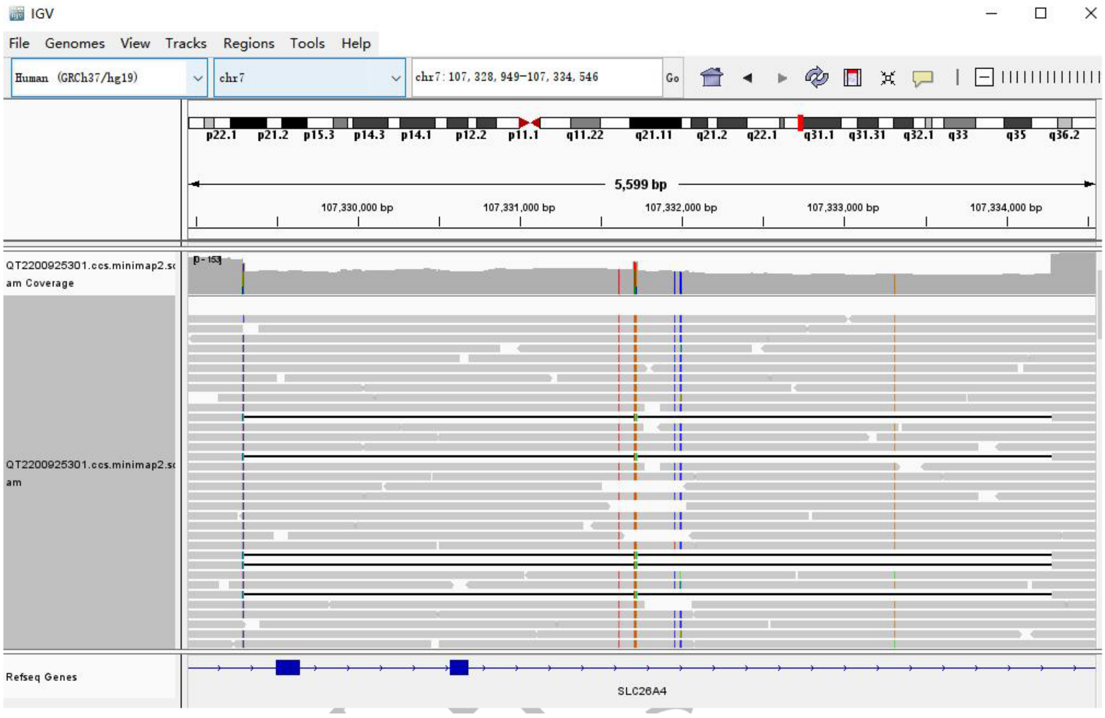

(D) Short version of Exons 1-3 deletion (3,152 bp)

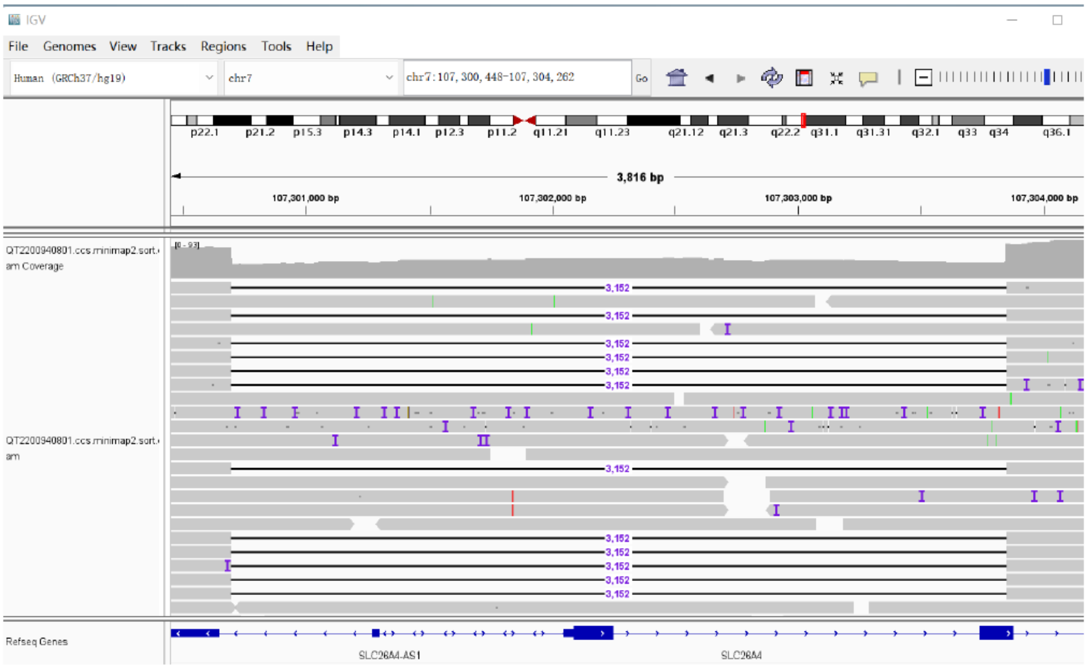
IGV
File Genomes View Tracks Regions Tools Help
Human (GRCh37/hg19)
chr7
chr7:107,300,448-107,304,262
Go
p22.1 p21.2 p15.3 p14.3 p14.1 p12.3 p11.2 q11.21 q11.23 q21.12 q21.3 q22.2 q31.1 q31.31 q32.1 q33 q34 q36.1
3,816 bp
107,301,000 bp
107,302,000 bp
107,303,000 bp
107,304,000 bp
QT2200940801.ccs.minimap2.sort.bam Coverage
QT2200940801.ccs.minimap2.sort.bam
3,152
Refseq Genes
SLC26A4-AS1
SLC26A4
